# Physicochemical and Sensory Characteristics of Instant Mushroom Soup Enriched with *Jerusalem artichoke* and Cauliflower

**DOI:** 10.3390/foods11203260

**Published:** 2022-10-18

**Authors:** Badr Saed, Mohammed El-Waseif, Hany Fahmy, Hamdy Shaaban, Hatem Ali, Manal Elkhadragy, Hany Yehia, Amr Farouk

**Affiliations:** 1 Food Science and Technology Department, Faculty of Agriculture, Al-Azhar University, Cairo 11651, Egypt; 2 Flavor and Aroma Chemistry Department, National Research Center, Cairo 12622, Egypt; 3 Food Technology Department, National Research Center, Cairo 12622, Egypt; 4 Department of Biology, College of Science, Princess Nourah bint Abdulrahman University, P.O. Box 84428, Riyadh 11671, Saudi Arabia; 5 Food Science and Nutrition Department, College of Food and Agriculture Science, King Saud University, P.O. Box 2460, Riyadh 11451, Saudi Arabia; 6 Food Science and Nutrition Department, Faculty of Home Economics, Helwan University, Helwan 11611, Egypt

**Keywords:** instant soup, mushroom, *Jerusalem artichoke*, *Cruciferous vegetables*, phytochemicals

## Abstract

The present study aimed to develop instant mushroom soup fortified with mixed *Jerusalem artichoke* and Cauliflower powders (JACF) instead of wheat flour at different levels (5, 10, 15, and 20%) based on dry weight as natural sources of protein, ash, fiber, inulin, and bioactive components. Based on the proximate analysis, adding JACF with 20% recorded the highest contents of protein, ash, fibers, and inulin as 24.73, 3.67, 9.67, and 9.17%, respectively. In the same line, macro- and microelements and essential amino acids showed a significant increase during fortification with 5–20% JACF compared to the control. In contrast, the total carbohydrate content and caloric values were decreased with the raised JACF concentration in the soup. The highest content of total phenolic acids, flavonoids, glucosinolates, carotenoids, and ascorbic acid was detected in mushroom soup with a 20% JACF mixture, which coincides with the highest antioxidant activity. Gallic (20.81–94.34 mg/100 g DW) and protocatechuic (13.63–58.53 mg/100 g) acids predominated among the phenolic acids identified in the mushroom-JACF soup samples, while rutin was the main flavonoid (7.52–18.2 mg/100 g). The increase of the JACF mixture in the soup significantly enhanced the rehydration ratio, total soluble solids, color parameters, and the sensory properties of the samples. In conclusion, using JACF in mushroom soup is necessary to improve the physicochemical characteristics and nutritional impact by containing phytochemicals and enhancing the organoleptic properties of the food product.

## 1. Introduction

In recent years, the tendency to consume instant foods has increased due to modern urbanization, increasing the number of working families, easy commercial availability, and minimally processed with longer shelf-lives up to 6 months at room temperature [1]. However, researchers’ efforts continue to improve the nutritional quality of instant soups by using vegetable resources of proteins, minerals, and vitamins suitable for vegetarians and others. The instant dry soup based on mushrooms represents one of the safest and most comprehensive foods appropriate for all age groups. As a nutrient-dense diet, mushrooms can be consumed in place of meat, fish, fruits, and vegetables [2]. It is a good source of protein; dietary fiber; minerals (P, K, Na, Ca, and Fe); vitamins (B1, B2, niacin, C, folic acid, and provitamin D ergosterol); and it is low in fat [3]. According to many studies [4], mushrooms have a protein content ranging from 20% to 40% on a dry matter basis and are rich in critical amino acids such as lysine and leucine that are scarce in cereal grains. Mushrooms were considered a therapeutic food due to their low content of fats; calories; sodium; [5] and presence of bioactive components, including polysaccharides, phenolics, glucans, terpenoids, and flavonoids [6], with their medicinal properties such as antioxidant, antimicrobial, anti-inflammatory, anticarcinogenic, hepatoprotective, antihyperglycemic, and anti-hypercholesteremia [7].

Previous studies dealt with fortifying mushroom instant dried soup with soy flour, moringa leaf, catfish oil microcapsules, legumes, and agro-wastes as sources for proteins, fibers, minerals, and other nutrients [2,8,9,10]. To our knowledge, nothing was reported concerning the supplementation of mushroom instant dry soup with vegetable resources rich in nutraceuticals such as inulin-type fructans with prebiotic activity or polyphenols. 

Inulin is used as a bioactive ingredient, mainly obtained from different parts of various plants belonging to the Asteraceae family, such as *Jerusalem artichoke* (*Helianthus tuberosus* L.) tubers containing 10–22 g of inulin/100 g fresh weight [11]. Other minority components of *Jerusalem artichoke* are proteins (2–3%), minerals (1–2%), and lipids (0.2–0.4%) expressed as %*w/w*. The countries where this ingredient is produced and those that must import it have seen an increase in the use of inulin in the food and beverage sectors, which has an impact on raising prices. The *Jerusalem artichoke* is a crop with competitive advantages that is also economically successful and a rich source of inulin, which can help meet the rising demand for this ingredient [11].

*Brassica* species are highly abundant in phytochemicals that promote health, such as phenolic compounds, vitamin C, and minerals. Protocatechuic acid (192.45), quercetin (202.4), pyrogallol (18.9), vanillic acid (11.90), coumaric acid (6.94), and kaempferol (25.91) mg/100 g DW were the major phenolics found in raw cauliflower, according to the HPLC examination [12]. Cauliflower is one of the vegetables rich in nutrients and excellent sources of protein (about 27% protein in dry matter), ash (11.03%); crude fiber (11.57%), and crude protein (27.77%), including amino acids such as methionine, phenylalanine, tyrosine and tryptophan, and total carbohydrates (47.43%) [12].

Therefore, based on *Jerusalem artichoke* (JA) and cauliflower (CF) rich in inulin and polyphenols, the present study aimed to improve instant mushroom soup by replacing wheat flour in the basic soup recipe with different levels (5, 10, 15, and 20%) of the JA and CF mixture as 1:1 (JACF). The physicochemical and sensory properties were examined to evaluate the fortified samples compared to the control. This study opens up prospects for adding nutraceuticals of vegetable origin that can promote health, are suitable for vegetarians, and are safe. 

## 2. Materials and Methods

### 2.1. Materials

Fresh, edible white mushrooms (*Agaricus bisporus* sp.) of the button variety were obtained from Oyster Company for Mushroom Production, Giza, Egypt. *Jerusalem artichoke* tubers (*Helianthus tuberoses* L.) were obtained from the Faculty of Agriculture, Ain Shams University. Cauliflower (*Brassica oleracea* var. *botrytis)*, wheat flour, skimmed milk powder, onion powder, garlic powder, and salt (sodium chloride) were purchased from the local market in Cairo, Egypt. All chemicals and reagents used in our study were analytical grade and obtained from El- Gamhouria Trading Chemicals and Drugs Company, Cairo, Egypt.

### 2.2. Methods

#### 2.2.1. Preparation of Mushrooms Powder

Fresh mushrooms were cleaned, washed with tap water, and sliced into small pieces 2 mm thick. Mushroom pieces were blanching in hot water at 60–70 °C for 2 min. Then, dried in an air-drying oven at 55 ± 2 °C for 24 h and grounded finely in a grinder (Braun JB3060 Tribute Collection Blender, Neu-Isenburg, Germany) into particles passing through 20-mesh sieves. The ground mixture is packaged in polyethylene bags and stored under cooling conditions (4 ± 1 °C). 

#### 2.2.2. Preparation of Mixed *Jerusalem artichoke* and Cauliflower Powders (JACF)

**Figure 1:**
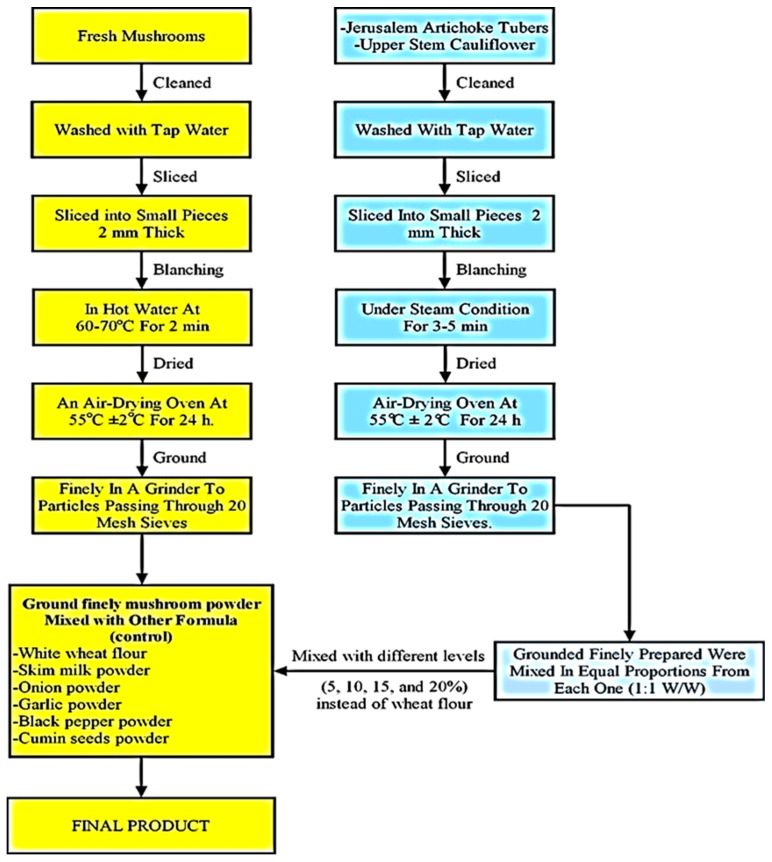
Flow diagram for the production of dried instant mushroom-JACF soup.

The whole *Jerusalem artichoke* tubers and upper stem cauliflower were washed with tap water, then cut into slices 2 mm thick, followed cooking under steam conditions for 3–5 min until soft. After drying in an air-drying oven at 55 ± 2 °C for 24 h, the whole *Jerusalem artichoke* and upper stem cauliflower were finely ground separately in a grinder into particles passing through a 20-mesh sieve. The ground finely prepared whole *Jerusalem artichoke* and upper stem cauliflower powders were mixed in equal proportions from each one (1:1 *w/w*), packaged by polyethylene bags, and stored under cooling conditions at 4 ± 1 °C (Figure 1). 

#### 2.2.3. Formulation of Dried Instant Mushroom-JACF Soup Samples

Five different blends, including a dried mushroom soup control sample (without JACF) and dried mushroom soup fortified with different levels (5, 10, 15, and 20%) of mixed *Jerusalem artichoke* and cauliflower powders (JACF) that were mixed in equal proportions (1:1 *w/w*), were prepared and are presented in Table 1 and Figure 1.

#### 2.2.4. Chemical Analysis of the Instant Soup Mixtures

The moisture, protein, fat, ash, and fiber of the dried mushroom soups were determined [13]. The total carbohydrates were calculated by difference. The amino acid profile of instant mushroom soup sample powders was measured according to Cosmos and Simon-Sarkadi [14] using an automatic amino acid analyzer AAA 400 (INGOS s.r.o., Prague, Czech Republic). Minerals (calcium, phosphorus, potassium, magnesium, sodium, iron, zinc, and copper) of different types of instant mushroom soup sample powders were determined according to the standard method of AOAC [13]. The energy values were calculated theoretically according to the method described by Paul and Southgate [15]. The inulin content of instant mushroom soup sample powders were measured according to Saengkanuk et al. [16]. The total glucosinolate content was determined as allyl isothiocyanate (mg/100 g dry weight basis) according to the method described by Mukhopadhyay and Bhattacharyya [17]. The total carotenoids were determined according to the procedure of Yuan et al. [18] at the absorbance of 451 nm. The total carotenoids were estimated by mg/100 g DW. Using the 2,6- dichloroindophenol titrimetric method, the ascorbic acid content was determined according to AOAC [13]. The ascorbic acid content is expressed as mg/100 g dry weight.

The antioxidant activity by DPPH (2,2-diphenyl-1picrylhydrazyl) free radical scavenging activity of the prepared soups was determined according to Ramesh and Satakopan [19]. The total polyphenols content was measured according to the modified Folin–Ciocalteu calorimetric method by Singleton et al. [20] at 760 nm using the digital spectrophotometer Spekol 11 (Carl Zeiss Spectroscopy GmbH, Jena, Germany) and expressed as gallic acid equivalent (GAE) per 100 g dry matter. The total flavonoid was determined according to the colorimetric method described by Bahorun et al. [21] at 510 nm using the digital spectrophotometer Spekol 11 (Carl Zeiss Spectroscopy GmbH, Jena, Germany), and the total flavonoids content was expressed as mg rutin equal/100 g dry weight basis. The identification of phenolic compounds was determined by using HPLC (Shimadzu, Kyoto, Japan) according to the method described by Goupy et al. [22].

#### 2.2.5. Physical Properties of the Prepared Soup Mixtures

A rehydration ratio test for instant mushroom soup sample powder was carried out, according to Krokida and Marinos-kouris [23]. The pH was determined as the method described in AOAC [13] by using a calibrated pH meter (Beckman 3550; Beckman Instruments Inc., Fullerton, CA, USA). Color values of the instant mushroom soup sample powders: L*, a*, and b* were determined by a colorimeter (CR-400, Konica Minolta, Inc., Osaka, Japan) [24].

#### 2.2.6. Sensory Evaluation of Instant Mushroom Soup Samples

An amount of 2.5 g of instant mushroom soup sample powder was dissolved in 25 mL boiling water and rehydrated for 2–3 min. The finished instant mushroom soup samples were subjected to a sensory assessment [25]. The sensory panel was composed of 4 women and 6 men, ranging in age from 22 to 30, and who were volunteer participants from the technical staff of the Department of Food Science and Technology, Faculty of Agriculture, Al-Azhar University. Ten panelists used a hedonic test 10-point scale to evaluate the organoleptic evaluation (color, taste, flavor, texture, odor, appearance, and overall acceptability), with 1 indicating extreme dislike and 5 indicating neither like nor a dislike, and 10 showing extreme like. All samples were analyzed in triplicate. The order in which the samples were presented was randomized.

#### 2.2.7. Statistical Analysis

All results were expressed as the mean values ± standard error by using a one-way analysis of variance (ANOVA) followed by the Duncan test using the SPSS statistical computer program.

## 3. Results and Discussion

### 3.1. Chemical Composition of Instant Mushroom Soup Samples

The results of the chemical compositions of the mushroom soup control and mushroom-JACF soup samples are shown in Table 2. It could be seen that the contents of crude protein, ash, crude fiber, and inulin in the prepared mushroom-JACF soup samples were significantly increased (*p* ˂ 0.05) by increasing the levels of added JACF from 5% to 20% in the mushroom soups. The obtained values of the crude protein content were significantly increased from 21.79 to 24.73 g/100 g, ash was 2.46 to 3.67 g/100, crude fiber was 8.44 to 9.67 g/100 g, and inulin 2.31 to 9.17 g/100 g compared with the control sample: 20.81, 2.75, 8.03, and 0 g/100 g, for protein, ash, crude fiber, and inulin, respectively. There were no significant differences *(p* ˂ 0.05) in the fat content by increasing the levels of added JACF in the mushroom-JACF soup samples: 3.70, 3.74, 3.77, and 3.81 g/100 g compared with the control sample (3.67 g/100 g). On the other hand, the other total carbohydrates content showed a significant decrease (*p* ˂ 0.05): 61.30 to 48.95 g/100 g, as well as caloric value; 368.54 to 340.47 kcal/100 g by increasing levels of added JACF from 5% to 20% in mushroom-JACF soup samples compared with control mushroom soup sample; (65.44 g/100 g for the other total carbohydrates and 378.03 kcal/100 g for the caloric value (Table 2). 

These increases in the crude protein, ash, crude fiber, and inulin contents of the prepared mushroom-JACF soup samples were remarkably due to the use of *Jerusalem artichoke* and cauliflower powders compared to wheat flour. In contrast, *Jerusalem artichoke* and cauliflower have very low other total carbohydrates compared to wheat flour (Table 2), which positively affects the caloric value of the total soup formula when the JACF mixture replaces wheat flour 5-20%. The effect of mushroom, *Jerusalem artichoke*, and cauliflower on the chemical composition of the total soup mixtures agrees with Roncero-Ramos et al., Ahmed and Ali; Yaseen et al., and Öztürk and Serdaroğlu [5,12,26,27] who reported the proximate compositions of the above plants.

In line with the studies focused on increasing the protein content of instant soups, the protein content of the presently developed mushroom-JACF mixture was higher than that of Farzana et al. [2], who supplemented mushrooms with soy and moringa with the highest protein content of 16.05%, and Mohamed et al. [8], who incorporated lyophilized chickpea, some vegetables, and some by-products (at 5% and 10%) in mushroom-based instant soup with 16.62–16.89% of the protein content. In contrast, Nguyen et al. [10] supplemented pearl oyster mushroom soup with some kinds of legumes and vegetables and reached a protein content of 25.60%, which is higher than the present study to a small extent. Well-researched studies show that dietary fiber is crucial for preventing several illnesses, including diverticulosis, constipation, irritable bowel syndrome, cancer, and diabetes [28]. Therefore, the soup powder that is now being created might help prevent certain disorders. In the same line, the crude fiber content in the present study was much higher than in other studies [2,29], which makes the created soup a fantastic fiber option. The ash content of the instant soup samples in the current study was found to be lower than the results of other studies, such as Singh et al. [30], who observed a 13.5% ash content in mushroom-whey soup powder, and Srivastava et al. [31] reported 12.00–16.35% ash in mushroom soups.

A low-fat diet can also help prevent dangerous illnesses such as diabetes, high cholesterol, and other disorders, including heart disease. The soup of the present study is a healthy option for everyone due to its decreased fat content compared to the literature results [2,8,30]. The energy of the prepared instant soups reported in Table 2 is comparable to the results of many authors, despite the contents of the formulas reported, which are related basically to reducing the total carbohydrates [2,8,31].

### 3.2. Mineral Contents of Instant Mushroom Soup Samples 

The macro- and microelements (K, P, Mg, Ca, Fe, Zn, Mn, and Cu) in the mushroom soup samples are listed in Table 3. By the addition of JACF from 5 to 20% in the mushroom soup, the increase in values of the minerals ranged from 1171.6 to 1412.75 mg/100 g for K, 447.48 to 535.50 mg/100 g for P, 76.23 to 122.72 mg/100 g for Mg, 109.02 to 145.98 mg/100 g for Ca, 4.85 to 6.60 mg/100 g for Fe, 2.78 to 3.43 mg/100 g for Zn, 1.52 to 1.91 mg/100 g for Mn, and 1.07 to 1.23 mg/100 g for Cu as compared with the control mushroom soup sample (1091.19, 418.27, 60.52, 96.66, 4.26, 2.56, 1.38, and 1.01 mg/100 g for K, P, Mg, Ca, Fe, Zn, Mn, and Cu, respectively (Table 3).

Replacing wheat flour with the JACF mixture in the mushroom-formulated soup led to an increase in minerals compared to the control sample. The higher content of minerals in the JACF mixture, according to Table 3, agreed with the investigations of Baloch et al. and Barkhatova et al. [32,33], who revealed higher micro- and macroelement contents in both cauliflower and *Jerusalem artichokes*. The above findings showed higher contents of minerals than the studies of Obiakor–Okeke et al. [34], Farzana et al. [2], and Mohamed et al. [8]. Mineral deficiencies are not associated only with malnutrition but also with many age-related disorders. Higher dietary Ca, Mg, K, and Fe intakes were associated with a lower risk of all-cause dementia, particularly vascular dementia, hypertension, stroke, Alzheimer’s neuropathology, cognitive function in elderly individuals, and anemia. Additionally, Zn improves mental health and immune function [35]. Consequently, based on the above analysis (Table 3), a serving of the mushroom soup samples (25 g/250 mL, *w/v*) containing 20% of the JACF mixture provided 7.51, 19.12, 7.30, 3.64, 26.12, 7.72, 20.43, and 33.33% of the RDI for K, P, Mg, Ca, Fe, Zn, Mn, and Cu, respectively.

### 3.3. Amino Acid Contents of Instant Mushroom Soup Samples

The essential and nonessential amino acid contents of the formulated prepared instant mushroom soup samples containing different levels of JACF are presented in Table 4. Among the essential amino acid contents, leucine (4.72 g/100 g sample), lysine (3.95 g/100 g sample), and the aromatic amino acids: phenylalanine and tyrosine mixture (3.42 g/100 g sample) were the highest recorded amounts and the predominates of the dried mushroom-JACF soup sample containing 20% JACF. On the other hand, histidine and isoleucine were the lowest in all the samples investigated (Table 4). Glycine, alanine, and arginine were the primary nonessential amino acids identified in all mushroom-JACF soups and the control. In contrast, glutamic acid was the lowest (Table 4).

The increase in the amino acid profiles during the addition of JACF mixture from 5 to 20% in the instant mushroom soup is due to the complete profiles of the essential and nonessential amino acids present in mushrooms, *Jerusalem artichokes*, and cauliflower [37,38,39]. Amino acids play numerous roles in the body, including oxygen carriers, neurotransmitters, raw materials for some hormones, and energy sources. In addition to helping to build cells and repair tissue, bone, skin, and muscle, amino acids are the primary building blocks for antibodies that fight off invading bacteria and viruses. These antibodies are a crucial component of the enzyme and hormone systems [37].

### 3.4. Phytochemicals in JACF and Mushroom-JACF Soup Samples

The phytochemicals, including the total polyphenolic acids, flavonoids, glucosinolates, carotenoids, and ascorbic acid, and the antioxidant activities were determined and are listed in Table 5. According to the obtained results, a significant increase (*p* ˂ 0.05) in JACF-mushroom soup samples by the addition of the JACF mixture from 5% to 20% could be observed (Table 5). Notably, phenolic compounds contribute directly to the antioxidant activity; consequently, it was necessary to investigate the total phenolic content. The obtained values of the total phenolic acid content significantly increased (*p* ˂ 0.05) from 151.22 to 580.33 mg/100 g with the addition of the JACF mixture from 5 to 20% (Table 5). Flavonoids have broad bioactivities, such as cell proliferation-inhibiting, apoptosis-inducing, enzyme-inhibiting, antibacterial, antioxidant, antiatherosclerosis, anti-inflammatory, antitumor, antithrombogenic, antiosteoporosis, and antiviral effects [40]. The total flavonoid content raised from 33.76 to 76.04 mg/100 g with the JACF addition compared to the control (9.89 mg/100 g) (Table 5). The previous findings agreed with cauliflower and *Jerusalem artichoke*’s higher phenolic acids and flavonoid contents. Ahmed and Ali [12] reported that the total of the phenolic acids and flavonoids in fresh cauliflower were 782.43 and 267.21 mg/100 g (on a dry weight basis), while they were 76.84 mg/100 g and 6.05 mg/100 gin *Jerusalem artichoke* tuber extracts [41].

Glucosinolates are a broad class of sulfur-containing molecules with anticancer action that is known to be the cause of the pungent flavor of the plants. They are one of the most significant phytochemicals in *Brassica* crops like cauliflower [42]. Therefore, it was expected to show an increase in the glucosinolates content from 123.40 to 505.94 mg/100 g during the JACF addition (Table 5). Plant pigments called carotenoids were detected in the mushroom-JACF soups with 22.49–48.27 mg/100 g found in the human diet as microconstituents of fruits and vegetables. The red, orange, and yellow colors of edible fruits and vegetables are caused by a class of aliphatic–alicyclic, fat-soluble chemicals also abundantly found in nature. They also have a protective role because of their conjugated double bonds, which can scavenge free oxygen radicals and reduce oxidative stress in organisms [43]. Fresh cauliflower is reported to have a high ascorbic acid content (769.23 mg/100 g on a dry weight basis) compared to *Jerusalem artichoke* (2.542 mg/100 g) according to Ahmed and Ali and Catană et al. [12,44]. Consequently, 5–20% of the JACF mixture detected 18.23–40.97 mg/100 g of ascorbic acid in the investigated instant soup samples (Table 5).

Antioxidant activities of the mushroom-JACF soup samples, as determined by the DPPH radical scavenging method, are shown in Table 5. In the DPPH scavenging assay, the antioxidant activity was measured by the decrease in absorbance as the DPPH radical received an electron or hydrogen radical from an antioxidant compound to become a stable diamagnetic molecule. DPPH radical scavenging activity expressed in % inhibition ranged from 25.20 to 70.22% based on the JACF amount added (5–20%) and compared to the control sample (11.3%). The previous findings agreed with the phenolic, flavonoid, and other phytochemical contents found in the current study. In the same line, raw and processed cauliflower extracts showed % inhibition of 35.13–68.91% [12], while the highest tested concentration of *Jerusalem artichoke* tuber extract exhibited an ability of radical scavenging over 56% [41]. It is noteworthy that the quantitative differences in phytochemicals and nutrients concentrations between the raw materials used in the current study and the literature are due to genetic and agronomical factors such as species, variety, climate, crop management strategies, postharvest storage, plant stage, and others [42].

### 3.5. Characterization of Phenolic Acids and Flavonoids Using HPLC

The HPLC analysis identified fifteen phenolic acids and four flavonoids in the JACF mixture and mushroom-JACF soup samples (Table 6). Gallic (20.81–94.34 mg/100 g DW) and protocatechuic (13.63–58.53 mg/100 g DW) acids were the predominant phenolic acids in soup samples during adding JACF (5–20%) compared to the control, which contains only 6.8 and 4.61 mg/100 g of gallic and protocatechuic acids (Table 6). Dramatically, the addition of JACF creates many other major phenolic acids compared to their concentrations in the control sample, such as pyrogallol (from 10.32 to 49.77 mg/100 g DW), p-hydroxybenzoic acid (from 3.17 to 47.01 mg/100 g DW), and p-coumaric acid (from 1.09 to 41.07 mg/100 g DW) (Table 6). On the other hand, cinnamic, vanillic, and 4-aminobenzoic acids remain minor among the other phenolic acids, despite the addition of the JACF mixture (Table 6). 

The previous findings are due to the higher phenolic acids content (2132.42 mg/100 g DW) of the JACF mixture used to supplement mushroom instant soup (Table 6). In agreement with the quantitative phenolic acids identified in the present study, and based on the JACF components, the leaves and seeds of cauliflower were reported to have hydroxybenzoic acid derivatives: gallic, protocatechuic, p-hydroxybenzoic, vanillic, and salicylic acid in the leaves, while gallic, protocatechuic, p-hydroxybenzoic, vanillic, syringic, and salicylic acid were identified in the seeds as the main phenolic acids [45]. On the other hand, *Jerusalem artichokes* have chlorogenic and caffeic acid derivatives as the major phenolic acids [46].

Rutin was the main flavonoid identified in all mushroom-JACF soup samples with 7.52–18.2 mg/100 g DW, followed by quercetin (7.11–16.44 mg/100 g DW) and kaempferol (6.21–16.19 mg/100 g DW) (Table 6). The same trend was shown in the control sample with 3.91, 2.47, and 2.13 mg/100 g DW for rutin, quercetin, and kaempferol, respectively. The JACF mixture is responsible for the increase of flavonoids in the mushroom-JACF soup samples, in addition to the appearance of rosmarinic acid with 7.07–12.7 mg/100 g DW, which was absent in the control sample (Table 6). Despite the quantitative differences, the previous findings agreed with the literature, where quercetin, rutin, kaempferol, and rosmarinic were the predominates among the flavonoids in cauliflower and *Jerusalem artichokes* [12,47]. The contents of the main bioactive substances in plants vary greatly based on geographic and environmental conditions, variety, growth stage, and harvest time. Additionally, the process conditions for extraction, such as extraction method, reagent, temperature, time, and solid-to-liquid ratio, also affect these factors [47].

Finally, the ability of mushroom-JACF soup recipes to scavenge DPPH radicals and their antioxidant activities highly correlates with their total phenolic contents. The phenolic acids and flavonoids have attracted considerable interest in the past few years due to their potential health benefits. They are potent antioxidants and have demonstrated antibacterial, antiviral, anticarcinogenic, anti-inflammatory, antioxidant, antitumor, and vasodilatory actions [48].

### 3.6. Physical Properties of Instant Mushroom-JACF Soup Samples

The physical properties of the instant mushroom soup samples supplemented with JACF (5–20%), including pH, rehydration ratio, total soluble solids, and color parameters, are listed in Table 7. No significant differences (*p* ˂ 0.05) in the pH values were observed among the mushroom-JACF soup samples compared to the control ones. On the other hand, the addition of JACF (5–20%) caused significant increases in all other physical parameters investigated in the current study (Table 7). 

In combination with proteins, dietary fibers provide high water-absorbing and water-holding abilities of food additives. Rehydration behavior, which significantly increased following the JACF addition, as shown in Table 7 (3.24–3.77 g), has been thought of as a mark of the material degradation caused by drying. A food product’s capacity to be reconstituted is primarily influenced by the internal structure of the dried pieces and the degree to which the water-holding elements (such as proteins and fibers) have been damaged during drying. This could be attributed to altered osmotic characteristics of the cell and decreased water diffusion through the surface during rehydration due to cellular structural damage. Better dried products with minimal changes to the structures of proteins or fibers and, consequently, minimal alterations to protein functioning are indicated by higher rehydration ratios [49].

According to Rubel et al. [11], fresh *Jerusalem artichokes* have a moisture content of 75–80% *w/w* and total carbohydrate content of up to 22%, with 70–90% inulin. The higher inulin content of *Jerusalem artichokes*, in addition to the soluble proteins and sugars of cauliflower, seem to be responsible for the significant increase in the total soluble solids of the JACF mixture and, consequently, the mushroom-JACF soups from 17.88 to 21.11% (Table 7). The number of fructofuranosyl units of inulin varies throughout the growing season, harvesting maturity and storage time after harvest. The inulin in mature tubers can contain 3–35 units; however, fructans with 2–10 units constitute the majority. Short-chain inulin with a polymerization degree <10 is water-soluble and has been used as alternative low-calorie sweeteners, while long-chain inulin with a polymerization degree >23 is less soluble and gives more viscous suspensions [11]. Soluble carbohydrates, besides inulin, are derivatives of fructooligosaccharides, reducing sugars: fructose, glucose, and sucrose. Soluble proteins and sugars were reported by Ahmed and Ali [12] and found to be lost either by leaching into the surrounding water or during the high temperature of stir-frying due to the Maillard reaction.

A significant increase could be observed in both color parameters a* (2.64–2.89) and b* (22.19–23.81), corresponding to the increase in redness and yellowness during the addition of JACF (5–20%). In contrast, L* values that correspond to lightness have no significant differences among the examined samples (Table 7). The increase of a* and b* occurs at the beginning of nonenzymatic browning due to the presence of high protein contents and sugars characterizing the JACF mixture. Despite the drying procedure and differences in the chemical compositions, the previous findings agreed with Catană et al. [44], who recorded 20.62 for b* and 3.14 for a* in convection-dried *Jerusalem artichokes*, and Sujatha et al. [50], who reported 8.8 and 28.5 for a* and b* in tray-dried cauliflower.

### 3.7. Organoleptic Evaluation of Instant Mushroom Soup Samples

The results of the organoleptic evaluation of all the prepared mushroom soup samples are presented in Table 8. The mushroom-JACF soup sample containing 20% JACF has the highest score of all the organoleptic parameters: color, taste, odor, flavor, texture, appearance, and overall acceptability: 7.89, 7.90, 7.91, 7.93, 7.77, 7.83, and 7.88, respectively, compared to the control sample: 7.31, 7.30, 7.29, 7.00, 7.29, 7.25, and 7.24, respectively. The previous findings are related to the significant increase (*p* ˂ 0.05) in the rehydration ratio (g); total solids soluble (%). and a* and b* values of the mushroom soup sample supplemented with 20% JACF. Additives like soy flour, moringa leaf, chickpea, vegetables, or agro-wastes may enhance the mushroom soup’s sensory properties, as shown by Farzana et al. and Mohamed et al. [2,8]. It seems that supplementation is essential not only from a nutritional and health point of view but also from the organoleptic side, as the increase in mushroom concentration in the instant soup formula from 10–40% may cause a decrease in the acceptability of the final product, as reported by Srivastava et al. [31]. 

## 4. Conclusions

The supplementation of mushroom soup with JACF seems essential not only for the health and nutrition impacts but also for the sensory properties of the food product. The addition of JACF mixture to mushroom instant soup at 5–20% instead of wheat flour led to an increase in the crude protein, crude fibers, ash, inulin, and essential amino acid elements, in addition to phytochemicals such as the total phenolics, flavonoids, glucosinolates, carotenoids, and ascorbic acid. Gallic and protocatechuic acids were the major phenolic acids, while rutin represented the main flavonoid identified in all the soup samples. The fortified mushroom instant soup sample with 20% JACF showed the highest sensory evaluation among the others.

## Figures and Tables

**Table 1:** Formulation of dried instant mushroom- JACF soup samples.

Ingredients (%)	Formulation of Dried Instant Mushroom-JACF * Soup Samples				
	Control	5% JACF	10% JACF	15% JACF	20% JACF
Dried mushroom powder	45	45	45	45	45
JACF *	-	5	10	15	20
White wheat flour	25	20	15	10	5
Skim milk powder	20	20	20	20	20
Onion powder	2.5	2.5	2.5	2.5	2.5
Garlic powder	2.0	2.0	2.0	2.0	2.0
Black pepper powder	1.5	1.5	1.5	1.5	1.5
Cumin seeds powder	1.0	1.0	1.0	1.0	1.0
Salt (sodium chloride)	3.0	3.0	3.0	3.0	3.0
Total	100	100	100	100	100

JACF *: Mixed *Jerusalem artichoke* and cauliflower powders in equal proportions (1:1 *w/w*).

**Table 2:** Chemical compositions of the mushroom-JACF * soup samples (on a dry weight basis).

Chemical Composition	Raw Materials			Mushroom-JACF Soup Samples (M ± SE)				
	WF	JA	CF	Control	5% JACF	10% JACF	15% JACF	20% JACF
Moisture (%)	9.11 ± 0.39 ^B^	9.44 ± 0.29 ^B^	8.95 ± 0.25 ^A^	9.94 ± 0.25 ^a^	9.81 ± 0.43 ^a^	9.79 ± 0.10 ^a^	9.76 ± 0.17 ^a^	9.74 ± 0.36 ^a^
Crude Protein (%)	7.68 ± 0.28 ^A^	9.11 ± 0.31 ^B^	28.89 ± 0.22 ^C^	20.81 ± 0.51 ^a^	21.79 ± 0.12 ^b^	22.77 ± 0.29 ^c^	23.75 ±0.14 ^d^	24.73 ± 0.08 ^e^
Ether extract (%)	1.74 ± 0.09 ^B^	1.54 ± 0.08 ^A^	3.97 ± 0.19 ^C^	3.67 ± 0.06 ^a^	3.70 ± 0.10 ^a^	3.74 ± 0.13 ^a^	3.77 ± 0.05 ^a^	3.81 ± 0.04 ^a^
Ash (%)	0.23 ± 0.39 ^A^	6.77 ± 0.27 ^B^	8.04 ± 0.30 ^C^	2.05 ± 0.06 ^a^	2.46 ± 0.10 ^b^	2.85 ± 0.02 ^c^	3.27 ± 0.04 ^d^	3.67 ± 0.11 ^e^
Crude Fiber (%)	0.22 ± 0.10 ^A^	4.53 ± 0.23 ^B^	12.91 ± 0.37 ^C^	8.03 ± 0.08 ^a^	8.44 ± 0.12 ^b^	8.83 ± 0.08 ^c^	9.26 ± 0.11 ^d^	9.67 ± 0.05 ^e^
Inulin (%)	-	75.17 ± 0.39	-	-	2.31 ± 0.10 ^a^	4.60 ± 0.10 ^b^	6.89 ± 0.10 ^c^	9.17 ± 0.10 ^d^
Other total Carp (%)	90.13 ± 0.47 ^C^	2.55 ± 0.11 ^A^	46.19 ± 0.22 ^B^	65.44 ± 0.12 ^e^	61.30± 0.42 ^d^	57.21 ± 0.18 ^c^	53.06 ± 0.55 ^b^	48.95 ± 0.85 ^a^
Caloric Value (kcal/100 g)	406.90 ± 0.79 ^C^	154.46 ± 0.44 ^A^	336.05 ± 0.51 ^B^	378.03 ± 0.51 ^c^	368.54 ± 0.51 ^bc^	359.33 ± 0.51 ^b^	349.78 ± 0.60 ^ab^	340.47 ± 0.59 ^a^

Data are expressed as the means ± SE; data on the same row with different superscripts were significantly different (*p* ˂ 0.05); Control mushroom soup. JA: *Jerusalem artichoke* powder. CF: cauliflower powder. WF: wheat flour. JACF *: Mixed *Jerusalem artichoke* and cauliflower powders mixed in equal proportions (1:1 *w/w*).

**Table 3:** Mineral contents (mg/100 g) of the instant mushroom soup samples (on a dry weight basis).

Samples		Minerals Content of JACF and Mushroom-JACF Soup Samples							
		K	P	Mg	Ca	Fe	Zn	Mn	Cu
JACF *		1577.12	579.59	319.88	258.77	11.53	4.20	2.71	1.17
control	DP *	1091.19	418.27	60.52	96.66	4.26	2.56	1.38	1.01
	Diluted N.S.S **	272.79	104.56	15.13	24.16	1.06	0.64	0.34	0.25
5% JACF	DP	1171.6	447.48	76.23	109.02	4.85	2.78	1.52	1.07
	Diluted N.S.S	292.90	111.87	19.05	27.25	1.21	0.69	0.38	0.26
10% JACF	DP	1251.97	476.89	91.75	121.37	5.42	3.04	1.63	1.13
	Diluted N.S.S	312.99	119.22	22.93	30.34	1.35	0.76	0.40	0.28
15% JACF	DP	1332.34	506.30	107.39	133.62	6.01	3.21	1.77	1.17
	Diluted N.S.S	333.08	126.57	26.84	33.40	1.50	0.80	0.44	0.29
20% JACF	DP	1412.75	535.50	122.72	145.98	6.60	3.43	1.91	1.23
	Diluted N.S.S	353.18	133.87	30.68	36.49	2.09	0.85	0.47	0.30
IMO ***		4.7 g/d	700 mg/d	420 mg/d	1000 mg/d	8 mg/d	11 mg/d	2.3 mg/d	0.90 mg/d

JACF: Mixed *Jerusalem artichoke* and cauliflower powders mixed in equal proportions (1:1 *w/w*). DP *: dried powder—diluted. N.S.S **: Normal serving size (25 g/250 mL water) *w/v*. IMO ***: Institute of Medicine of the National Academies [36].

**Table 4:** Amino acid (g/100 g sample) contents of JACF and mushroom-JACF soup samples.

Amino Acids	JACF *	Mushroom-JACF Soup Samples				
		Control	5% JACF	10% JACF	15% JACF	20% JACF
Histidine	0.39	1.41	1.39	1.38	1.37	1.35
Lysine	1.82	3.59	3.68	3.75	3.84	3.95
Valine	1.83	2.37	2.46	2.54	2.6	2.69
Leucine	1.21	4.48	4.54	4.61	4.66	4.72
Isoleucine	1.19	1.61	1.67	1.75	1.81	1.9
Threonine	1.58	1.78	1.86	1.91	1.98	2.1
Methionine + Cysteine	8.36	0.85	1.27	1.66	2.11	2.41
Phenylalanine + Tyrosine	2.82	2.87	3.01	3.14	3.3	3.42
Total Essential Amino acids	19.2	18.96	19.88	20.74	21.67	22.54
Arginine	0.39	0.35	0.37	0.38	0.4	0.41
Alanine	0.42	0.41	0.44	0.46	0.47	0.52
Glycine	0.37	0.6	0.62	0.63	0.64	0.66
Aspartic acid	0.43	0.19	0.2	0.22	0.23	0.24
Glutamic acid	0.21	0.11	0.12	0.13	0.13	0.14
Total non-Essential Amino acids	1.82	1.66	1.75	1.82	1.87	1.97
Total Amino acids	21.02	20.62	21.63	22.56	23.54	24.51

JACF *: Mixed *Jerusalem artichoke* and cauliflower powders mixed in equal proportions (1:1 *w/w*).

**Table 5:** Phytochemicals and antioxidant activity of JACF and mushroom-JACF soup samples.

Bioactive Components	JACF *	Mushroom-JACF Soup Samples (M ± SE)				
		Control	5%JACF	10% JACF	15% JACF	20% JACF
Total phenolic acids	2301.18 ± 1.76 ^a^	34.12 ± 0.70 ^a^	151.22 ± 0.75 ^b^	268.22 ± 0.77 ^c^	390.31 ± 0.77 ^d^	580.33 ± 0.75 ^e^
Total flavonoids	487.93 ± 0.90 ^a^	9.89 ±0.44 ^a^	33.76 ± 0.47 ^b^	50.08 ± 0.45 ^c^	62.11 ± 0.45 ^e^	76.04 ± 0.51 ^d^
Glucosinolates(mg/100 g)	2393.89 ± 1.90	-	123.40 ± 0.25 ^a^	247.65 ± 0.25 ^b^	370.2 ± 0.25 ^c^	505.94 ± 0.25 ^d^
Carotenoids (mg /100 g)	219.12 ± 0.78	11.24 ± 0.25 ^a^	22.49 ± 0.25 ^b^	30.3 ± 0.25 ^c^	39.74 ± 0.25 ^d^	48.27 ± 0.25 ^e^
Ascorbic acid (mg /100 g)	159.77 ± 0.44	10.11 ± 0.25 ^a^	18.23 ± 0.25 ^b^	26.83 ± 0.25 ^c^	33.0 ± 0.25 ^d^	40.97 ± 0.25 ^e^
DPPH (%)	117.23 ± 0.49	11.3± 0.22 ^a^	25.20 ± 0.21 ^b^	47.90 ± 0.22 ^c^	63.12 ± 0.21 ^d^	70.22 ± 0.25 ^e^

Data are expressed as the means ± SE; data in the same row with different superscripts were significantly different (*p* ˂ 0.05). JACF *: Mixed *Jerusalem artichoke* and cauliflower powders mixed in equal proportions (1:1 *w/w*).

**Table 6:** HPLC analysis of the phenolic compounds (mg/100 g DW) of the JACF and mushroom-JACF soup samples.

Phenolic Compounds	JACF *	Mushroom-JACF Soup Samples				
(mg/100 g)		Control	5%JACF	10% JACF	15%JACF	20% JACF
	**Phenolic acids**					
Gallic acid	279.87	6.8	20.81	39.01	60.76	94.34
Protocatechuic	179.12	4.61	13.63	28.71	38.89	58.53
Pyrogallol	178.44	1.31	10.32	20.11	37.89	49.77
p-hydroxybenzoic	221.31	3.17	14.16	25.07	31.8	47.01
Caffeic	199.13	3.9	14.11	23.78	30.08	42.18
p-Coumaric acid	189.77	1.09	12.11	23.25	30.01	41.07
Ellagic	81.6	1.22	5.7	11.09	23.33	39.17
Catechin	191.43	4.11	15.22	22.03	30.56	39.11
Alpha-coumaric	178.87	1.08	10.11	15.07	21.09	34.13
Coumarin	119.77	1.22	7.3	11.66	19.03	27.9
Chlorogenic	99.71	0.77	5.13	9.07	14.13	20.03
Catechol	77.91	0.65	4.86	9.01	15.12	19.8
Cinnamic acid	61.23	0.4	3.3	7.05	10.12	15.01
Vanillic	31.12	0.32	1.96	4.02	9.7	11.75
amino benzoic	43.13	0.31	2.88	6.13	8.07	11.23
Total polyphenol acids	2132.41	30.96	141.6	255.06	379.95	551.03
	**Flavonoids**					
Rutin	88.41	3.91	7.52	11.88	14.11	18.2
quercetin	93.57	2.47	7.11	11.03	13.38	16.44
kaempferol	81.65	2.13	6.21	10.11	13.18	16.19
Rosmarinic	141.33	-	7.07	9.03	10.9	12.7
Total Flavonoids	404.96	8.51	27.91	42.05	51.57	63.53
Total Phenolic compounds	2537.37	39.47	169.51	297.11	431.52	614.56

JACF *: Mixed *Jerusalem artichoke* and cauliflower powders mixed in equal proportions (1:1 *w/w*).

**Table 7:** Physical properties of the instant mushroom soup samples.

Physical Properties		JACF **	Instant Mushroom Soup Samples				
			Control	5%JACF	10%JACF	15% JACF	20%JACF
pH value		7.10 ± 0.05	7.09 ± 0.09 ^a^	7.11 ± 0.08 ^a^	7.12 ± 0.07 ^a^	7.12 ± 0.09 ^a^	7.13 ± 0.09 ^a^
Rehydration ratio (g)		4.52 ± 0.20	3.02 ± 0.23 ^a^	3.24 ± 0.26 ^b^	3.49 ± 0.19 ^c^	3.61 ± 0.25 ^d^	3.77 ± 0.25 ^d^
Total solids soluble (%)		34.99 ± 0.28	16.12 ± 0.31 ^a^	17.88 ± 0.31 ^b^	18.94 ± 0.28 ^c^	20.07 ± 0.32 ^d^	21.11 ± 0.32 ^d^
Color	L^*^	72.68 ± 0.25	76.03 ± 0.20 ^a^	75.17 ± 0.19 ^a^	74.79 ± 0.18 ^a^	74.05 ± 0.20 ^a^	73.30 ± 0.20 ^a^
	a^*^	3.88 ± 0.19	2.59 ± 0.16 ^a^	2.64 ± 0.15 ^a^	2.70 ± 0.15 ^ab^	2.79 ± 0.17 ^bc^	2.89 ± 0.17 ^c^
	b^*^	23.44 ± 0.21	21.38 ± 0.19 ^a^	22.19 ± 0.16 ^ab^	22.51 ± 0.17 ^b^	23.16 ± 0.15 ^bc^	23.81 ± 0.15 ^c^

Data are expressed as the means ± SE; data in the same row with different superscripts were significantly different (*p* ˂ 0.05). JACF **: Mixed *Jerusalem artichoke* and cauliflower powders in equal proportions (1:1 *w/w*).

**Table 8:** Organoleptic properties of the instant mushroom soup samples.

Organoleptic Properties	Instant Mushroom Soup Samples (M ± SE)				
	Control	5%JACF *	10% JACF	15% JACF	20% JACF
Color	7.31 ± 0.33 ^a^	7.46 ± 0.35 ^ab^	7.71 ± 0.33 ^bc^	7.82 ± 0.30 ^bc^	7.89 ± 0.31 ^c^
Taste	7.30 ± 0.30 ^a^	7.50 ± 0.29 ^ab^	7.70 ± 0.28 ^bc^	7.84 ± 0.31 ^bc^	7.90 ± 0.30 ^c^
Odor	7.29 ± 0.29 ^a^	7.51 ± 0.25 ^ab^	7.70 ± 0.33 ^bc^	7.83 ± 0.27 ^bc^	7.91 ± 0.27 ^c^
Flavor	7.00 ± 0.31 ^a^	7.52 ± 0.35 ^b^	7.73 ± 0.29 ^b^	7.86 ± 0.28 ^bc^	7.93 ± 0.30 ^c^
Texture	7.29 ± 0.30 ^a^	7.49 ± 0.26 ^ab^	7.61 ± 0.33 ^ab^	7.70 ± 0.27 ^b^	7.77 ± 0.29 ^b^
Appearance	7.25 ± 0.33 ^a^	7.43 ± 0.35 ^ab^	7.64 ± 0.33 ^bc^	7.71 ± 0.29 ^bc^	7.83 ± 0.27 ^c^
Overall acceptability	7.24 ± 0.30 ^a^	7.47 ± 0.33 ^ab^	7.68 ± 0.29 ^bc^	7.79 ± 0.27 ^bc^	7.88 ± 0.31 ^c^

Data are expressed as the means ± SE; data in the same row with different superscripts were significantly different (*p* ˂ 0.05). JACF *: Mixed *Jerusalem artichoke* and cauliflower powders in equal proportions (1:1 *w/w*).

## Data Availability

The data are available from the corresponding author.
